# Performance assessment of a multi-epitope chimeric antigen for the serological diagnosis of acute Mayaro fever

**DOI:** 10.1038/s41598-021-94817-x

**Published:** 2021-07-28

**Authors:** Paloma Napoleão-Pêgo, Flávia R. G. Carneiro, Andressa M. Durans, Larissa R. Gomes, Carlos M. Morel, David W. Provance, Salvatore G. De-Simone

**Affiliations:** 1 Oswaldo Cruz Foundation (FIOCRUZ), Center for Technological Development in Health (CDTS), National Institute of Science and Technology for Innovation in Diseases of Neglected Populations (INCT-IDPN), Brazil Av 4365, Leonidas Deane Building, Room 309, Rio de Janeiro, RJ 21040-900 Brazil; 2 Laboratory of Interdisplinary Medical Research (LIPMED), Oswaldo Cruz Institute (IOC), FIOCRUZ, Brazil Av 4365, Rio de Janeiro, RJ 21040-900 Brazil; 3 Biology Institute, Federal Fluminense University, Outeiro de São Joao Batista S/N, Niterói, RJ 24020-141 Brazil

**Keywords:** Immunology, Microbiology, Molecular biology

## Abstract

Mayaro virus (MAYV), which causes mayaro fever, is endemic to limited regions of South America that may expand due to the possible involvement of *Aedes *spp. mosquitoes in its transmission. Its effective control will require the accurate identification of infected individuals, which has been restricted to nucleic acid-based tests due to similarities with other emerging members of the Alphavirus genus of the Togaviridae family; both in structure and clinical symptoms. Serological tests have a more significant potential to expand testing at a reasonable cost, and their performance primarily reflects that of the antigen utilized to capture pathogen-specific antibodies. Here, we describe the assembly of a synthetic gene encoding multiple copies of antigenic determinants mapped from the nsP1, nsP2, E1, and E2 proteins of MAYV that readily expressed as a stable chimeric protein in bacteria. Its serological performance as the target in ELISAs revealed a high accuracy for detecting anti-MAYV IgM antibodies. No cross-reactivity was observed with serum from seropositive individuals for dengue, chikungunya, yellow fever, Zika, and other infectious diseases as well as healthy individuals. Our data suggest that this bioengineered antigen could be used to develop high-performance serological tests for MAYV infections.

## Introduction

Mayaro virus (MAYV) is an emerging zoonotic circulating arbovirus that is part of the *Alphavirus* genus and *Togaviridae* Family. Due to its antigenic nature, it is included in the Semliki Forest group. First isolated in Mayaro, Trinidad and Tobago in 1954^1,2^, its current geographical distribution exclusively encompasses Central and South American countries, mainly those containing extensive areas of tropical forests^3–9^. MAYV was first isolated in Brazil in 1955, and since then, it has been the cause of several epidemics in the North and Central-West regions, where it can be considered endemic^10–13^. The most recent incidence was in a cluster of individuals from a rural village of Venezuela infected in 2010^14^.

Its primary vector is the Haemagogus janthinomys mosquito; however, other mosquito genera are also known as vectors, such as *Culex* and *Psorophora*^3,15^. Some studies suggest that *Aedes aegypi* is another potential vector, which would be considerably troublesome to public health. Not only because this mosquito is well adapted to the urban environment, there would also be a possibility to associate MAYV dissemination to the spread of dengue virus (DENV)^16,17^. The recent demonstration that MAYV can infect multiple species of *Anopheles* mosquitoes in laboratory settings raises concerns for the spread of disease beyond its current geographical distribution to other continents^18^.

The MAYV genome is composed of a single positive RNA chain consisting of 11,429 nucleotides. Viral particles are enveloped with a diameter of 69 ± 2.3 nm and an icosahedral nucleocapsid^19^. The first Open Reading Frame (ORF), starting from the 5′ end, encodes a polyprotein that undergoes cleavage during the translation process and generates four non-structural proteins (nsP1, nsP2, nsP3, and nsP4)^20,21^. A second ORF region is contained in the 3′ end that, when translated, produces subgenomic RNA 26S and, sequentially, into a structural polyprotein that yields E1, E2, E3, 6K proteins, and capsid (C) proteins^22–24^.

MAYV is an arbovirus of great concern due to the high rate of morbidity observed with infection^25^, which can cause a highly debilitating clinical condition, Mayaro fever, characterized by high fever, headaches, diarrhea, vomiting, skin eruptions, myalgia, and persistent arthralgia^14,26,27^. Its symptoms are similar to those from other arboviruses that co-circulate in areas endemic for MAYV that include DENV, chikungunya (CHIKV), yellow fever (YFV), and oropouche (OROV) that can lead to a clinical misdiagnosis of MAYV infections for one of them^28–30^. Correct diagnoses of MAYV infections are further complicated by its short viremic phase^17^, which compounds the difficulty of a molecular diagnosis by directly detecting viral nucleic acid by reducing the virus's capture in biological samples obtained from clinically symptomatic patients. Lastly, the laboratory techniques developed for an alphavirusis plaque assay are complicated^31^, which has limited its implementation as a routine diagnostic assay.

The Global Research Collaboration for Infectious Disease Preparedness has highlighted the need for the urgent development of specific diagnostic assays for the widespread surveillance of MAYV, CHIKV, and O'nyon-nyong virus (ONNV), which has been pointedly discussed by the international GloboPid-R group^32^. A variety of diagnostic tests have been proposed based on molecular assays^12,33^, ELISAs using viral proteins^34,35^, and ELISAs based on chimeric alphavirus proteins^36^. For serological diagnostic assays, the cross-reactivity of antibodies generated from related alphavirus antigens, such as CHIKV and others, is a significant concern^37–39^. Consequently, new high throughput methodologies must be applied to identify and exclude these cross-reactive sequences during the development of new immunological diagnostic tests.

Here, we report on our innovative approach to generate highly specific and sensitive immunoreagents for serodiagnostic assays that begins with a search for pathogen specific epitopes in the B-cell epitome of a virus. For MAYV, four epitopes were identified based on binding to Mayaro fever patient IgM antibodies that originated from the nsP1, nsP2, E1, and E2 proteins from MAYV. Two copies of three of the epitopes along with three copies of the other epitope were fused in silico into specific positions of a synthetic gene encoding for a β-barrel protein (eGFP) that can support the insertion of extraneous sequences without compromising its core structure. Termed Dx-MAYV-M, the chimeric multi-epitope protein was expressed in an *Escherichia coli* system, purified, and evaluated as an in-house ELISA antigen to detect IgM antibodies in serum from patients confirmed with a MAYV infection. As negative controls, a panel of patient sera infected with DENV, CHIKV, YFV, and zika (ZIKAV) viruses and other infectious diseases such as hepatitis B, leptospirosis, leishmaniasis, and Chagas disease were analyzed and its performance compared to the use of individual epitopes in the form of multiple antigen peptides in peptide ELISAs.

## Results

### Epitope selection

**Figure 1:**
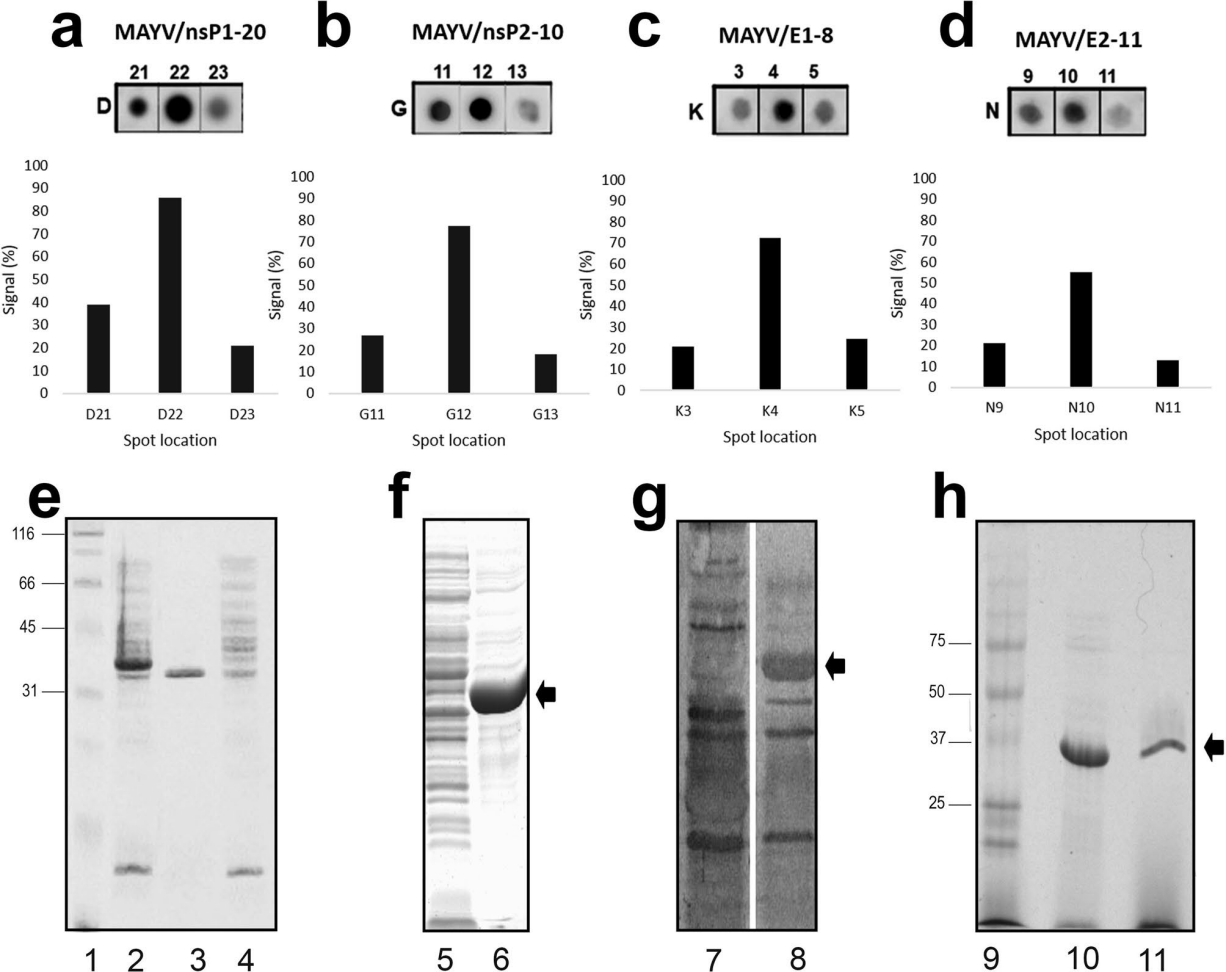
Epitope identification and production of Dx-MAYV-M. The entire IgM epitome of MAYV was revealed by Spot synthesis. The image of the reactivity from three consecutive peptides that define the minimal motif of the four epitopes that showed the lowest theoretical potential for cross-reactivity to other pathogens, along with a quantification of the relative intensity, is indicated for nsP1-20 (**a**); nsP2-10 (**b**); E1-8 (**c**); and E2-11 (**d**). Overexpression of the bioengineered Dx-MAYV-M protein in *E. coli* led to mostly insoluble protein that could be purified by IMAC. (**e**) SDS-PAGE with lane 1, molecular markers; lane 2, whole extract; lane 3, inclusion bodies; lane 4, soluble proteins. (**f**) An overloaded SDS-PAGE gel of soluble (lane 5) and insoluble (lane 6) protein. (**g**) A western blot of soluble (lane 7) and insoluble (lane 8) protein probed with Mayaro fever patient sera. (**h**) SDS-PAGE of Dx-MAYV-M before (lane 9) and after (lane 10 and 11) affinity purification by a HisTrap column (from insoluble fraction and soluble proteins, respectively).

A complete Spot-synthesis analysis identified 134 linear B-cell IgM epitopes that were recognized by a pool of patient sera (N = 5 of 18) in the five structural (C, E1, E2, E3, and 6K) and four non-structural (nsP1, nsP2, nsP3, nsP4) MAYV proteins. As a primary goal of this work was to identify antigenic determinants that could differentiate MAYV infections from closely related pathogens, bioinformatics was used to BLAST the sequence of each of the 134 epitopes. Four epitopes that originated from proteins nsP1, nsP2, E1, and E2 were determined to meet the criteria for a high potential to avoid cross-reactivity based on the absence of multiple sequential amino acids were identical to segments in other pathogens (Table 1), including the most highly similar virus, Una Virus^19^. Figure 1a–d shows the image of the four-peptide spots that encompass the individual peptides along with a graphical representation of their relative intensities. From the four groups, the minimal motifs comprising the epitope nsP1-20 (^466^HRIRLLLQS^474^), nsP2-10 (^240^NGVKQTVDV^248^), E2-11 (^619^SYRTFGAERV^628^), and E1-8 (^1010^QSRTL DS^1016^) were chosen for further analysis.

**Table 1:** Selected epitopes mapped in MAYV proteins using a pool of sera from patients with mayaro fever.

aa	Epitope sequence	Code	Insertion site of eGFP (aa)
466–474	HRIRLLLQS	MAYV/nsP1-20huM	1,2; 186,187; 253,254
240–248	NGVKQTVDV	MAYV/nsP2-10huM	127,128; 225,226
619–628	YRTFGAER	MAYV/E2-11huM	112,113; 202,203
1010–1018	SRTLDSR	MAYV/E1-8huM	148,149; 165,166

### Dx-MAYV-M production

Each antigenic region was bioengineered into the backbone of eGFP into two independents sites, except HRIRLLQS; three copies were inserted. Table 1 lists the amino numbers of the insertion sites performed in silico to create the multi-epitope chimeric recombinant protein referred to as Dx-MAYV-M. It was produced as a synthetic gene for cloning into pET-28a that maintained an amino terminus six-histidine affinity tag while excluding the tag available on the carboxy terminus. The final protein had a deduced molecular mass of 40.6 kDa with an isoelectric point of 8.35. Within the pET expression system, the recombinant protein was readily overexpressed in *E. coli* (~ 250 µg/ml) and was localized to inclusion bodies when the expression temperature was 37 °C (Fig. 1e). An increased proportion of soluble protein was observed at lower expression temperature, albeit at lower levels. In addition to the higher production levels, the use of inclusion bodies provided an initial purification step resulting in > 90% purity that also avoided degradation of Dx-MAYV-M during bacteria lysis and avoided the need for protease inhibitors (Fig. 1f).

A western blot of the soluble and insoluble recombinant protein probed with a pool of serum from patients with Mayaro fever confirmed that Dx-MAYV-M was recognized by anti-MAYV antibodies (Fig. 1g). Some other bands could be observed to be attributed to human sera's general reactivity against bacterial proteins. The purification of Dx-MAYV-M by immobilized metal affinity chromatography (IMAC) was feasible in the presence of urea, and soluble protein could be purified after removal of urea on-column before elution (Fig. 1h).

### ELISA-peptide assay and performance comparison

**Figure 2:**
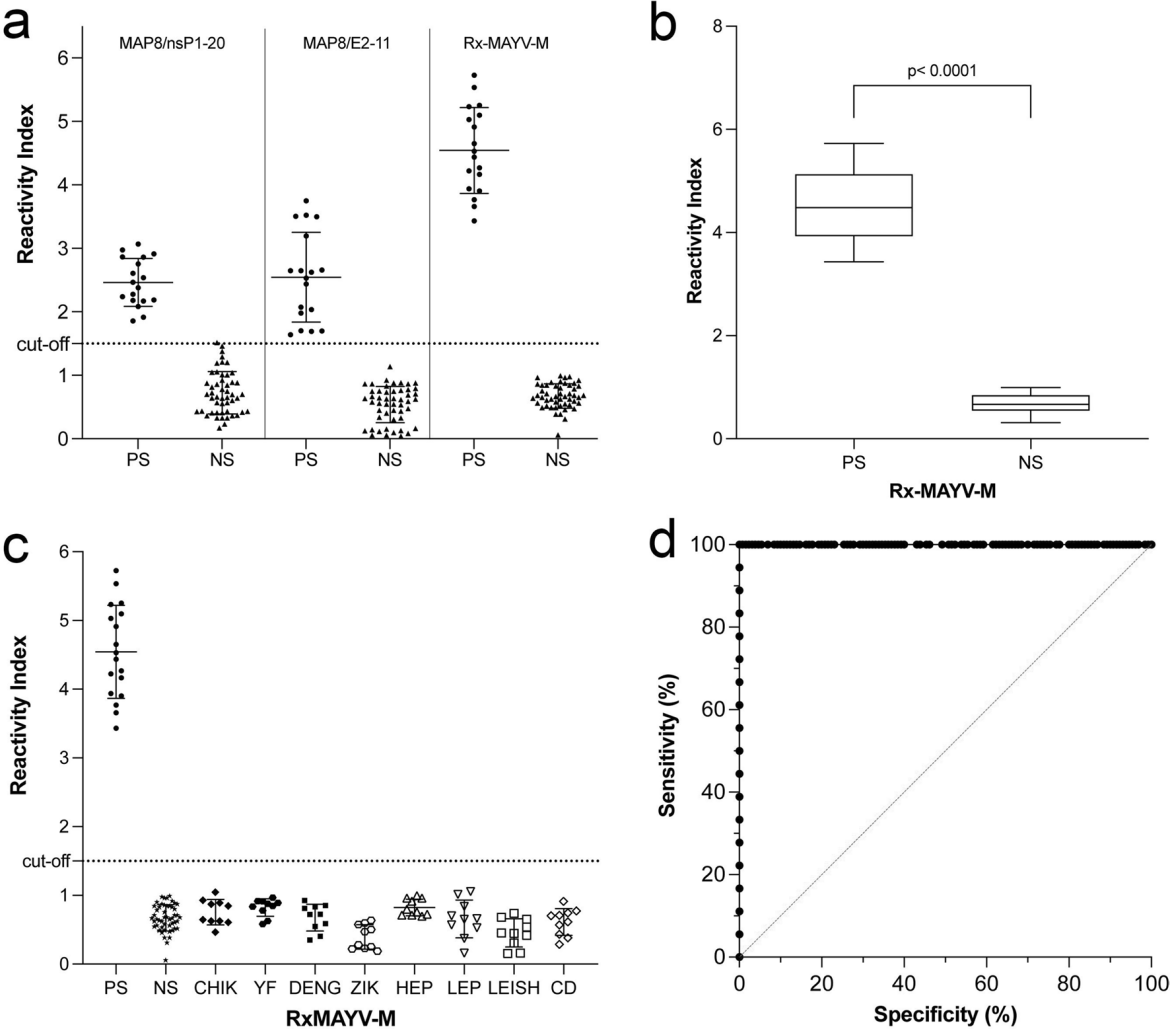
Diagnostic performance of IgM epitopes in MAYV as peptides and in Dx-MAYV-M. In-house ELISAs were generated with peptides MAP8/nsP1-20 (RIRLLLQGGNGVKQT VD) and MAP8/E2-11 (YRTFGAERGGSRTLDSR), and the engineered Dx-MAYV-M. (**a**) Their reactivity index for each of 18 serum samples from Mayaro fever patients, 80 serum of other infectious diseases [CHIKV, YFV, ZIKV, HBV, LEP, LEISH, CD]. (**b**) The median and 95% confidence interval, along with maximum and minimum indices of IgM reactivity of Dx-MAYV-M, was significant (Brown–Forsythe and Welch, p = 0.0001). (**c**) The absence of cross-reactivity by antibodies against a panel of serum positive for other pathogens co-circulating with Dx-MAYV-M (PS, MAYV positive; NS, negative healthy individuals; CHIKV, Chikungunya; YFV, Yellow fever; DENV, Dengue; ZIKV, Zika; HBV, Hepatitis B; LEP, Leptospirosis; LEISH, Leishmaniosis; and CD, Chagas disease). (**d**) ROC curve analysis for Rx-MAYVM.

To verify the epitopes' expected individual diagnostic performance, two synthetic bispecific multiple antigen peptides with eight copies (MAP8) were chosen and used in an in-house peptide ELISA. The particular serum in the pool of the five from infected patients used in the peptide library screen along with an additional thirteen was tested for sensitivity, and a variety of sera from patients infected with CHIKV, YFV (vaccinated), DENV, ZIKV, hepatitis B (HBV), leptospirosis (LEP), leishmaniasis (LEISH), and Chagas disease (CD) patients along with fifty, non-symptomatic healthy donors were tested for cross-reactivity. Excellent performance for MAYV/nsP1-20 and MAYV/E2-11 with all MAYV patient serum and signals above the cut-off was measured (Fig. 2a). Besides, no cross-reactivity was observed, and the results were statistically significant (p < 0.001).

### Performance of Dx-MAYV-M in Mayaro fever's serodiagnosis

To determine whether the multi-epitope chimeric protein could be considered an effective tool for the serodiagnosis of acute MAYV infections, we analyzed its reactivity against the same panel of sera as used to screen the peptide library (Fig. 2a). The positive reactivity indices for MAYV patient serum increased more than twofold in comparison to the MAPs. As importantly, the reactivity indices for the negative controls were lower, which gave a large statistically significant difference, p < 0.0001, between positive and negative signals that could significantly reduce borderline results (Fig. 2b). Considering the high number of infectious agents that co-circulate in endemic regions of MAYV, serum from another eighty individuals with other diseases were evaluated, and the reactivity against each of the pathogens was grouped (Fig. 2c). Based on a Receiver Operating Characteristics (ROC) curve (Fig. 2d), the area under the curve (AUC) for MAYV-M protein varied from 0.9839 to 0.9998 (p < 0.0001) as detected by ELISA with interval confidence of 95%, demonstrating high diagnostic accuracy for the Dx-MAYV-M recombinant chimeric proteins (Supplementary Figures S1–S6).

### Spatial localization of the epitopes in Dx-MAYV-M

**Figure 3:**
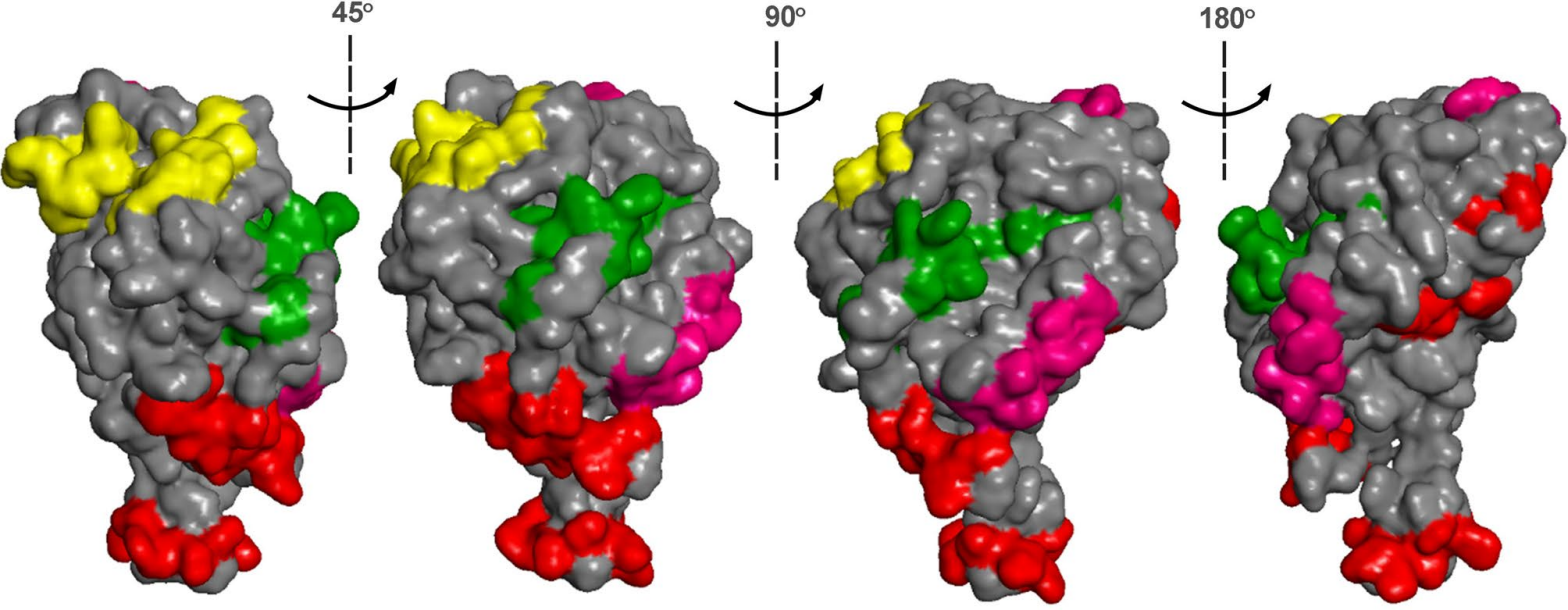
Spatial localization of the MAYV IgM linear epitopes in Dx-MAYV-M. A space-filling model of a computer-predicted structure for the multi-epitope chimeric protein with the core structure of the eGFP in gray along with the epitope of nsP1-20 in red, nsP2-10 in yellow, E1-8 in pink, and E2-11 in green. Multiple angles are depicted.

The I-Tasser server generated a predicted structure of the entire Dx-MAYV-M protein that presented a confidence score equal to − 1.41 and a TM-score of 0.47 ± 0.15 (borderline quality). The epitope sequences' localization on the chimeric protein's surface was confirmed using a generated space-filling model. Figure 3 shown the individual epitopes colored. All four of the selected linear epitopes were located to surface regions in Dx-MAYV-M. A hydropathy plot also suggested that all of the epitopes were present on the proteins' surface (data not shown).

## Discussion

Emerging viral diseases cause great concern due to their potential to spread undetected well beyond their geographical origins. In many instances, early symptoms are indistinguishable from those associated with known endemic viruses, especially related family members. Also, definitive diagnostic tests most likely do not exist or require time and extensive laboratory-based techniques are cannot be feasibly applied on a large scale for implementation.

This fact is undoubtedly the case for MAYV. As an arbovirus, the range of competent mosquito vectors raises the possibility that human travel could spread the disease to multiple continents, including North America, Africa, and Asia^18^. To date, no rapid, specific diagnostic test has been developed.

The diagnosis of a MAYV-infection depended principally on identifying the virus by its genome's molecular detection. A hemagglutination inhibition (HI) assay was developed^40,41^ but has been mostly abandoned due to the assay's low sensitivity. Attempts have been made to improve serodiagnostic assays using recombinant proteins representing viral proteins as antigens to capture IgG antibodies in in-house ELISAs^38,40^. However, these assays' results have displayed considerable variations in their reproducibility, reliability, feasibility, and specificity, with significant cross-reactivity being described, which is a substantial challenge in the field for the differential diagnosis of highly similar pathogens.

Here, we focused on detecting MAYV specific IgM antibodies as their production can begin between 3–8 days after infection^42,43^. This timing overlaps with the onset of clinical symptoms (3–5 days post-infection) and could be detectable over the course of a hospitalization, which can last more than five days. As the levels of IgM antibodies generated against them often reach detectable levels during the associated illness, in general, this is consistent with the alphaviruses' observations.

We also considered that a serologically based assay's performance is directly related to the target's capacity in a diagnostic test to bind pathogen-specific antibodies, which are then detected by the labeled, secondary antibody that can differ depending on the platform. Reliance on viral proteins to serve as the target cannot deliver the performance profile for specificity and sensitivity for an effective diagnostic and surveillance tool. While the immune system's capacity to generate antibodies against a pathogen is the keystone of serodiagnosis, it is, in fact, a mixture of antibodies that range from specific to non-specific and high to low reactivity. Ultimately, the characteristics of an antibody involve its cognate epitope sequence in the proteome of the pathogen, including an understanding of the biochemical and antigenic characteristics of the epitopes that interact with the antibodies, which are crucial to controlling factors during an assay that can affect the antigen–antibody interaction, such as ionic strength and pH as well as the formation of aggregates of the target in the assay solvent^44,45^.

Therefore, to develop a diagnostic assay for detecting anti-MAYV IgM, we began with identifying the antigenic determinants within the proteome of MAYV based on the identification of its linear B-cell epitopes for IgM. Thus, as the successful binding of antibodies to proteins has been correlated with hydrophilicity and the surface exposure of epitopes^45,46^, we used characteristics to reduce the primary list of epitopes. Next, we restricted the epitopes based on the highest affinity according to their relative signal strength in the Spot Synthesis analysis. Finally, to minimize the potential for cross-reactivity, the epitope sequences were subjected to a BLAST homology analysis to identify those strictly associated with the MAYV proteome and no others (data not shown). At the end of our investigation, only 4 of the 134 epitopes identified were selected. Importantly, these epitopes were from four different proteins and strengthened the argument that the naturally occurring organization of epitopes within the proteome of a pathogen is not optimal for developing diagnostic tests.

As the availability and spatial distribution of the epitopes in a target on the solid phase of the test can influence performance, linearized epitopes as peptides have the advantage of presenting individual fragments separately for their interaction with polyclonal antisera that avoids overlapping to minimize steric hindrance competition. To evaluate multiple antigen peptide format, two epitopes of the four epitopes, as shown in Fig. 2. The results showed excellent specificity that showed that peptides nsP1-20 and E2-11 are not found in any other pathogen as expected from the sequence analysis. While a separation in the signal pattern between positive samples and the negative controls was observed, the use of a diagnostic assay at the earliest times of IgM production required the highest possible sensitivity. We chose to combine the four different IgM linear B-cell epitopes into a single chimeric protein.

Combining multiple epitopes into a continuous poly amino acid chain successfully produced as recombinant protein is complex in practice. First, most epitopes are found at the surface of a protein and must remain there in the chimeric protein to maintain their capacity to interact with antibodies. The simple stringing together of multiple epitope sequences with interspersing amino acids does not guarantee this organization, nor does it provide an interior to the resulting protein. Secondly, the final structure is thoroughly unpredictable due to the complexity of protein folding. To resolve these issues, we searched for a naturally occurring protein whose structure is well defined and understood, as well as has extensive experimental data that shows its capacity for withstanding alterations without compromising its ability to be expressed.

Our search led to the identification of eGFP as an excellent candidate to serve as a proteinaceous receptacle to receive a large number of extraneous epitope sequences. The prominent defining structural feature of eGFP in the β-can formed by 11 discrete β-sheet segments^47^. Multiple experiments have shown that the formation of the β-can is responsible for most of the biochemical properties such as rate of folding, aggregation, and stability^47–49^. The changes in multiple, specific amino acids in the β-sheets that form the barrel's staves altered these properties. Conversely, the exact sequence of the intervening regions appeared unimportant in the generation of an expressable protein. Several examples exist in the literature for the insertion/replacement of up to three of the sites^50–54^. However, this is the first demonstration for the replacement of an extreme number. A significant advantage of our approach was the availability of templates for computer modeling.

During the chimera multi-epitope protein construction, the select highly immunogenic sequences were reconsidered and the most hydrophilic epitope sequences for the sequence replacement location. Before generating the synthetic gene, the coding sequence of the bioengineered Dx-MAYV-M multi-epitope protein was subjected to a computer simulation to predict the spatial positioning of the introduced epitope sequences to consider that the substitution/ insertion of peptide sequences into the protein matrix could lead to the concealment or impediment of the accessibility of these sequences to the surface of the molecule and hence to the antibodies^55^. In addition to being localized on the surface, each epitope was depicted as a random coil structure, an excellent structure for interacting with its cognizant antibody and should improve the potential for high diagnostic accuracy.

The chimeric protein maintained the excellent specificity of the MAPs to detect IgM antibodies only in the sera from patients confirmed to be infected by MAYV. The ELISA results using the Dx-MAYV-M recombinant chimeric multi-epitope protein revealed that all epitopes appropriately discriminated between negative and positive samples (p < 0.0001). No cross-reactivity was observed as anticipated from the BLAST analysis criteria. Compared to the individual epitope MAPs, the multi-epitope protein's use improved our in-house ELISA's performance by generating a higher signal that showed a tremendous difference between positive and negative samples. From the ROC analysis, the sensitivity of the ELISA was 99.6%. This increase in movement most likely reflects the presence of multiple copies of each epitope and their incorporation into a protein environment instead of being peptides. Another benefit of the multi-epitope protein design was to increase the specific activity of each molecule to bind antibodies, with each containing up to nine sites.

Phase I studies are typically conducted using a group of seropositive individuals and seronegative individuals to assess whether the test can differentiate between them^56^. For MAYV, the sample size of the positive samples is still tiny, primarily due to the difficulties for its diagnosis that prevent infected patients' identification during the disease's acute phase. This fact limits the scope of the likelihood score of the ROC analysis that relates to the probability of an increase in sample size to alter the assay's sensitivity and specificity. A much more extensive and diverse panel of negative controls was available to support our conclusion that Dx-MAYV-M has 100% specificity. The performance to date strongly supports the continued use of Dx-MAYV-M as a target in diagnostic tests and suggests that the antigen may be eligible to enter phase II studies.

## Methods

### Serum samples and ethical considerations

This investigation was approved by the Ethical Committee of FIOCRUZ (CEP/IOC-CAAE:52892216.8.0000.5248 and 384/07) following the principles of the Declaration of Helsinki. The voluntary donors provided informed consent by a signed Free and Informed Consent Form (TCLE) for themselves or as the adult responsible for a minor without a definition of their health status, social group, age, sex, or race. The sera of patients with Mayaro fever used in the study (N = 18) were provided by the Leonidas and Maria Deane Institute (FIOCRUZ-Amazonas), FIOCRUZ-Rondônia and Evandro Chagas Institute. Patient serum was initially identified by characteristic signs of mayaro fever (chills, fever, gastrointestinal manifestations, eye pain, myalgia, and arthralgia) considered positive by an in-house agglutination test and confirmed by RT-PCR^32^. All symptoms appeared between 2 and 5 days, except for arthralgia. Various laboratories provided eighty other serum samples from patients with different pathologies (CHIKV, YFV (vaccinated), ZIKV, HBV, LEP, LEISH, and CD) at the Oswaldo Cruz Institute, Rio de Janeiro, Brazil, and 40 healthy individuals by blood bank donors of Rio de Janeiro (HEMORIO).

### Epitope mapping

The complete sequences of non-structural (Q8QZ72) and structural (Q8QZ73) proteins of MAYV circulating in Brazil were obtained through access to the Uniprot database (http://www.uniprot.org/). Microarrays of peptides were used to map linear B-cell epitopes, as described previously^57^. One hundred thirty-four linear B-cell IgM epitopes were identified using a pool of a random subset (N = 5) of patient sera infected with MAYV (non-published data).

### Synthetic gene, protein expression, and purification

Two copies of three epitopes and three copies of one epitope sequences were inserted into the green fluorescent protein (GFP, *Aequorea victoria*) core protein sequence^47^ as described in Table 1. The final chimeric amino acid sequence was back transcribed into an optimized nucleic acid for expression in bacteria with end restriction sites for cloning into pET28a, including an amino terminus 6xHis tag. A synthetic gene fragment (GeneArt, ThermoFisher) was cloned in a single step into the expression vector, and the resulting clone was confirmed by sequencing. The plasmid was transformed into *Escherichia coli* BL21 (DE3) by standard techniques for recombinant protein expression. Production was induced for 3 h at 37 °C with 0.8 mM of isopropyl β-d-1-thiogalactopyranoside. Cells were collected by centrifugation and disrupted by sonification in lysis buffer (300 mM NaCl, 50 mM Tris, pH 8.0). Inclusion bodies were collected by centrifugation, washed in lysis buffer with 0.5% Triton-X100, and resuspended in lysis buffer with 6 M urea and 20 mM imidazole. Overnight (at 4 °C), solubilized proteins were clarified by centrifugation, and Dx-MAYV-M was purified by affinity chromatography on a HisTrap Q HP column (GE Healthcare, Piscataway, NJ) connected to an Aktä prime chromatography system (GE Healthcare, Piscataway, NJ). Urea was removed by a gradient wash followed by a two-step elution with 250 and 500 mM imidazole. Fractions with Dx-MAYV-M, as confirmed by SDS-PAGE**,** were diluted and applied to the HiTrap column to remove imidazole and concentrate the chimeric protein. The final Dx-MAYV-M preparation was quantified spectrophotometrically and confirmed by SDS-PAGE.

### Synthesis of MAPs containing bispecific-peptides

For the preparation of the two MAP8 peptides, a standard solid-phase synthesis protocol was used with the octameric Fmoc8-Lys4-Lys2-Lys-B-Ala Wang resin that provides eight reaction sites as described previously^58^. Each peptide epitope [nsP1-20 (RIRLLLQGGNGVKQ TVD) and E2-11 (YRTFGAERGGSRTLDSR)] was linked in tandem with the addition of two extra Gly residues to improve the presentation of antigens to antibodies. Briefly, the construct was prepared in an automated peptide synthesizer (PSS8-model, Shimadzu, Kyoto, Japan), and the side chains of octafunctional Fmoc-amino acids were protected with trifluoracetic-labile protecting groups as required. Residues corresponding to the monovalent ('tail') part of the construct, up to the first (bis-Fmoc) Lys residue initiating the dendrimer structure, were incorporated via single couplings. Once Fmoc groups were removed and sequence assembly completed, the peptide-resin was cleaved and fully deprotected with TFA/H2O/EDT/TIS (94/2.5/ 2.5/1.0 v/v, 90 min). The peptides were precipitated by the addition of chilled diethyl ether followed by centrifugation, then the resulting pellet was taken up into aqueous AcOH (10% v/v), dried, and stored as a lyophilized powder. When necessary, the MAP was dissolved in water, centrifuged (10,000*g*, 60 min, 15 °C) and the supernatant filtered by a centricon filter. Synthetic peptides (MAP8/nsP1-20 and MAP8/E2-11) were purified by high-performance liquid chromatography (HPLC) using a Hi-Pore RP318 column and characterized by mass spectrometry at the National Institute of Quality Control on Health (INCQS) of FIOCRUZ, Rio de Janeiro, Brazil.

### In-house ELISA optimization and procedure

Optimal target quantities along with dilution of patient serum and secondary antibody were determined by checkerboard titration. After optimization, Dx-MAYV-M chimera was used at 300 ng/well in coating buffer (0.05 M carbonate-bicarbonate, pH 9.6) to sensitize high-binding 96-well microplates (Jet Biofil, Guangzhou, China) overnight at 4 °C. Microplates were rinsed and blocked with TBSM (TBS with 1% fat-free dry milk) for 1 h at 37 °C and washed with TBST before adding patient serum samples (100 μl, diluted 1:150 in TBS). Plates were incubated for 90 min at 37 °C, washed in TBST, and then incubated with alkaline phosphatase-conjugated goat anti-human IgM (1:20,000) for 90 min at 37 °C. Next, plates were washed five times before the addition of 100 μl of PNPP (p-nitrophenyl phosphate, 1 mg/ml, Thermo Fisher, USA). After a 15 min incubation at room temperature in the dark, the absorbance at 405 nm was measured in a FlexStation-3 microplate reader (Molecular Devices, San José, CA, U.S.A.).

### Molecular modeling

As three-dimensional structure data does not exist for Dx-MAYV-M, a theoretical model from the primary sequence of the chimeric protein was generated using the I-TASSER server (http://zhanglab.ccmb.med.umich.edu/I-TASSER/), which combines the methods of threading, ab initio modeling, and structural refinement^59^. A model with a TM-score value greater than 0.70 and a reasonable quality c-score was chosen for visualization using Visual Molecular Dynamic (VMD) 1.9^60^.

### Statistical analysis

Data were encoded and analyzed using scatter computer graphic software (GraphPad Prism version 6, San Diego-CA, USA). Descriptive statistics were presented as geometric mean ± SD. Reactivity performance of the enzyme-linked immunosorbent assay was evaluated by receiver operating characteristic curve (ROC) curve analysis. The ROC curve also produces a table related to specificity, sensitivity, and cut-off (cut-off). The cut-off selection criteria were 100% specificity and 100% sensitivity. The D'Agostino and Pearson test and Kologorov-Smirnv test, followed by Student's *t* test, were used to confirm the normality of datasets and the variance homogeneity assumption. For analysis of variance with confidence intervals, the ANOVA (Brown–Forsythe and Welch) parametric test wax was used. This index is referred to as reactivity index (RI), and all results < 1.00 were considered harmful. However, samples were deemed inconclusive (or in the gray zone) if the RI values fell into the undetermined zone, hypothesized as RI values of 1.0 ± 10%.

## Supplementary Information

Supplementary Figures.

## Data Availability

The full dataset that supports the findings of this study is available from the authors upon reasonable request.
