# Supplementary material for: Performance assessment of a multi-epitope chimeric antigen for the serological diagnosis of acute Mayaro fever
Source: Sci Rep. 2021 Jul 28;11:15374. doi: 10.1038/s41598-021-94817-x (PMC8319364; doi:10.1038/s41598-021-94817-x)
Supplement: Supplementary file 1 — Supplementary Figures. [file 41598_2021_94817_MOESM1_ESM.pdf]

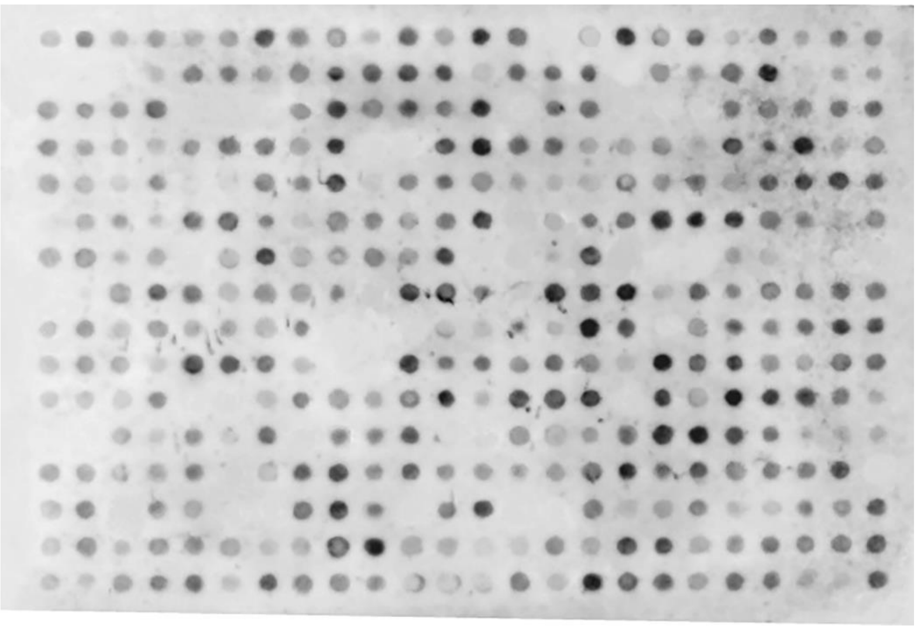

Supplementary Figure S1: Cellulose membrane, showing the synthetic peptides of several MAYV proteins, reactive to pool of sera of patients with Mayaro fever through the technique of Spot synthesis, revealed with anti-human IgM alkaline phosphatase. Those of interest for this work are shown in detail in Figure 1a.

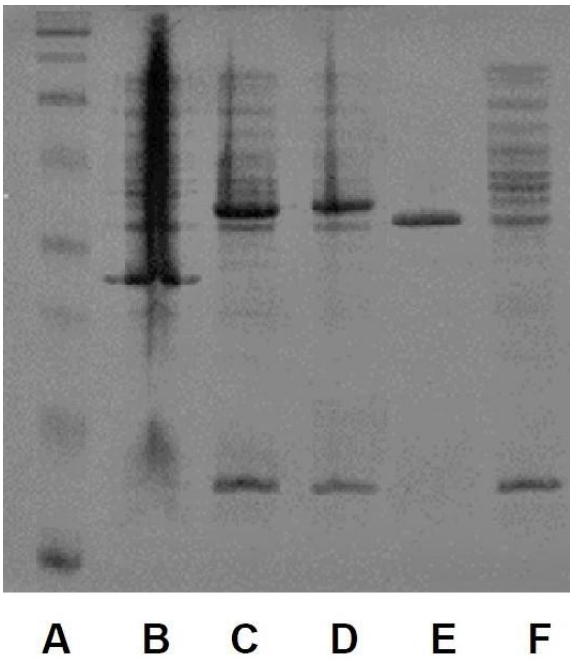

Supplementary Figure S2: Expression of the recombinant protein Dx-MAYV-M analyzed by SDS-PAGE (12%). (A) Standard molecular weight proteins; (B) Total protein extract from the Rx platform (control); (C) Total extract; (D) Insoluble fraction (inclusion bodies); (E) Soluble fraction**.**

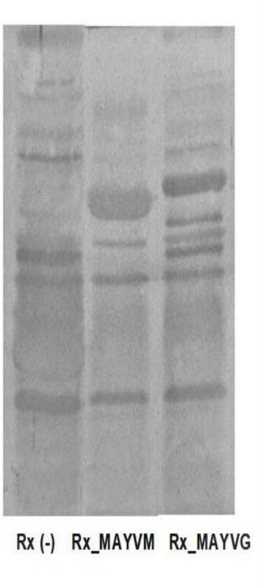

Supplementary Figure S3: Western blotting of the recombinant protein Dx-MAYV-M present in the insoluble fraction (inclusion bodies extracted with sample buffer) and serum from patients with mayaro fever (n = 10). The lane on the left side represents the protein Dx-without the MAYV epitopes, and on the right side, another protein distinct from Dx-MAYV-M cloned and analyzed by the same technique.

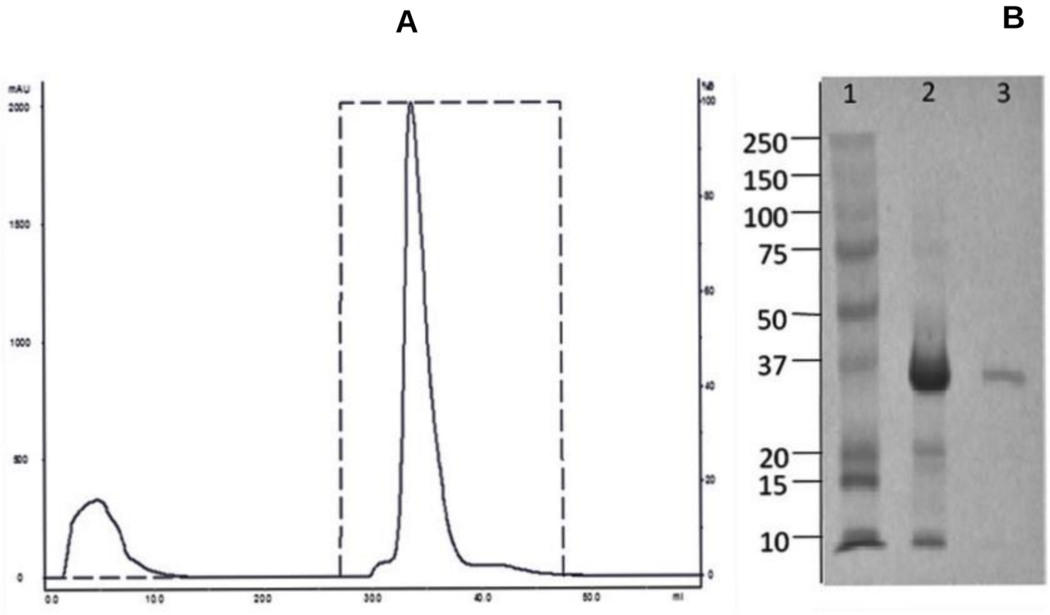

Supplementary Figure S4: Purification of the recombinant protein Dx-MAYV-M by affinity chromatography (Hitrap HP, 5mL) using an Aktä high-performance liquid chromatography system (A) and analysis by SDS-PAGE (B, 12%). The column was equilibrated in TU buffer (20mM Tris-HCl, pH 8.0, containing 8M urea), and elution was performed in TUI buffer (TU containing 500 mM imidazole). B: SDS-PAGE (12%): (1) Molecular weight standard; (2) insoluble fraction Dx-MAYV-M; (3) Purified Dx-MAYV-M protein**.**

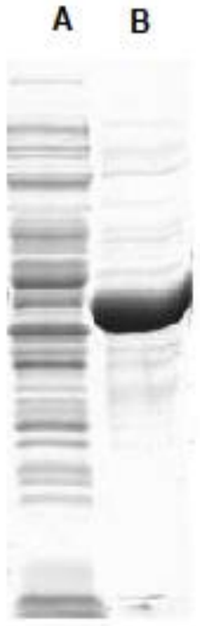

Supplementary Figure S5: SDS-PAGE (12%) of the cloned Dx-MAYV-M recombinant protein. (A) Soluble fraction, (B) insoluble fraction Dx-MAYV-M.

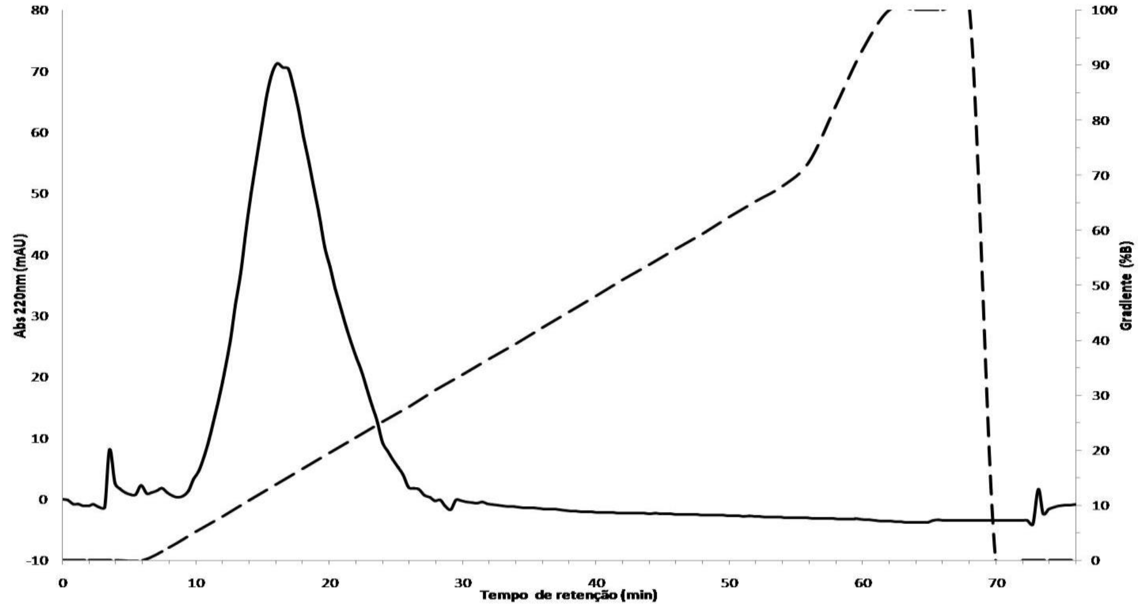

Supplementary Figure S6: Purification of the MAP8-synthetic peptide (MAYV/nsP1-20) through a Hi-Pore RP318 column (10 x 250 mm). The eluents were: (A) 0.1% TFA in H2O; (B) 80% acetonitrile containing 0.7% TFA. The solid line represents the profile at 220nm, the dashed line the gradient of eluent B**.**
